# SonoBox: development of a robotic ultrasound tomograph for the ultrasound diagnosis of paediatric forearm fractures

**DOI:** 10.3389/frobt.2024.1405169

**Published:** 2024-08-21

**Authors:** Floris Ernst, Jonas Osburg, Ludger Tüshaus

**Affiliations:** ^1^ Institute of Robotics and Cognitive Systems, University of Lübeck, Lübeck, Germany; ^2^ Department of Paediatric Surgery, University Hospital Schleswig-Holstein, Lübeck, Germany

**Keywords:** children, forearm fracture, ultrasound robot, radiation reduction, contactless imaging

## Abstract

**Introduction:**

Paediatric forearm fractures are a prevalent reason for medical consultation, often requiring diagnostic X-rays that present a risk due to ionising radiation, especially concerning given the sensitivity of children’s tissues. This paper explores the efficacy of ultrasound imaging, particularly through the development of the SonoBox system, as a safer, non-ionising alternative. With emerging evidence supporting ultrasound as a viable method for fracture assessment, innovations like SonoBox will become increasingly important.

**Materials and methods:**

In our project, we want to advance ultrasound-based, contact-free, and automated cross-sectional imaging for diagnosing paediatric forearm fractures. To this end, we are building a technical platform that navigates a commercially available ultrasound probe around the extremity within a water-filled tank, utilising intelligent robot control and image processing methods to generate a comprehensive ultrasound tomogram. Safety and hygiene considerations, gender and diversity relevance, and the potential reduction of radiation exposure and examination pain are pivotal aspects of this endeavour.

**Results:**

Preliminary experiments have demonstrated the feasibility of rapidly generating ultrasound tomographies in a water bath, overcoming challenges such as water turbulence during probe movement. The SonoBox prototype has shown promising results in transmitting position data for ultrasound imaging, indicating potential for autonomous, accurate, and potentially painless fracture diagnosis. The project outlines further goals, including the construction of prototypes, validation through patient studies, and development of a hygiene concept for clinical application.

**Conclusion:**

The SonoBox project represents a significant step forward in paediatric fracture diagnostics, offering a safer, more comfortable alternative to traditional X-ray imaging. By automating the imaging process and removing the need for direct contact, SonoBox has the potential to improve clinical efficiency, reduce patient discomfort, and broaden the scope of ultrasound applications. Further research and development will focus on validating its effectiveness in clinical settings and exploring its utility in other medical and veterinary applications.

## 1 Introduction

### 1.1 Robotics in paediatric medical care

In modern medicine, robotic devices have started to play a role in the early 1990s with systems such as RoboDoc (Paul et al., 1992) and the CyberKnife (Adler et al., 1997). In the years since, medical robotics have become an integral part of many clinical processes, covering such different areas as surgery (Lauterbach et al., 2017), radiotherapy (Gerlach and Schlaefer, 2022), disinfection (Mehta et al., 2023), and others. Still, these systems are mostly extremely expensive and their medical value is sometimes disputed (Barbash Gabriel and Glied Sherry, 2010). Even worse, most robotic systems are not tailored for use with paediatric patients, thus excluding this patient group from potential technological benefits. We believe that the ongoing technological push, together with the unmet clinical needs and the current health economic challenges (e.g., shortage of health workers), should be seen as a great opportunity for innovation especially for children (Dimitri et al., 2021).

In particular, AI applications, medical robotics, additive manufacturing with 3D printing technology, and miniaturization show promising developments in basic technologies that enable novel medical technology solutions. Medical robotics in particular benefits synergistically from these technologies. In order to enable efficient medical technology development with successful subsequent transfer into clinical care, close cooperation between doctors, engineers, computer scientists, parent initiatives and medical device companies is mandatory.

We have been investigating these issues (Tüshaus et al., 2018), as well as possible fields of clinical healthcare where robotics could improve paediatric care (Großbröhmer et al., 2023), and have come to the conclusion that ultrasound robotics could play a pivotal role in improving care for the diagnosis of paediatric fractures.

### 1.2 Use case

Forearm fractures and forearm contusions are very common causes of medical presentation in children and adolescents (Kraus and Wessel, 2010). As part of standard diagnostics, an X-ray examination is often performed in two planes, whereby inconspicuous findings are also very frequently obtained (this generally applies to approx. 80% of all X-ray examinations performed). Due to the increased sensitivity of children’s tissue to radiation, it is desirable to reduce or eliminate the risk of exposure to ionising X-rays following the ALARA (as low as reasonably achievable) principle (Kraus and Dresing, 2023). Children must be protected from X-rays and, where possible, procedures without ionising radiation should be used for imaging. Interestingly, 80% of all x-rays show normal findings for all age groups, so that a fracture can be ruled out (Brooks et al., 1981). Current evidence supports bone sonography as a substitute for X-ray examination:

1. Ultrasound is a safe, fast, side-effect-free and health-economically relevant point-of-care (PoC) diagnostic technique that is available in many cases (Ackermann et al., 2024).
2. Current studies show a clear advantage for the risk assessment of a possible fracture with ultrasound imaging and examination according to defined quality standards by experienced physicians (Ackermann et al., 2019).

In fact, comparable sensitivity and specificity for ultrasound and X-ray as imaging modalities could be shown for different pediatric fracture types (Ackermann et al., 2024; Snelling et al., 2023). Nevertheless, the decisive argument is the lack of radiation exposure of fracture ultrasound.

**FIGURE 1:**
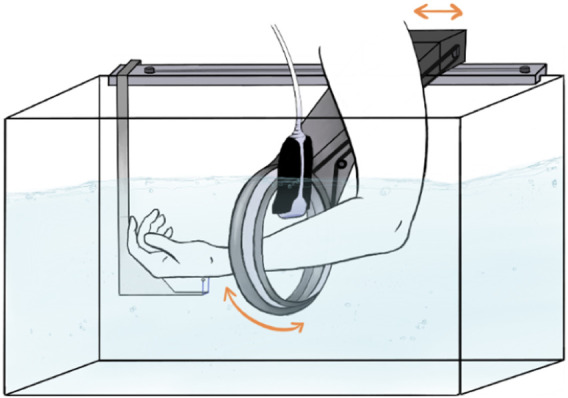
A concept sketch of the envisioned SonoBox device. Figure reproduced with permission of the author Annika Dell, MSc, Fraunhofer Research Institution for Individualized and Cell-Based Medical Engineering, Lübeck, Germany.

We believe that, for this paediatric scenario, a robotic ultrasound system where a large 3D ultrasound scan of the patient’s forearm can be acquired, would be the ideal solution. A concept sketch is shown in Figure 1.

### 1.3 Clinical background

Bone fractures and contusions of the forearm are among the most common accidents in childhood. According to (Kraus, 2016), the overall fracture risk, the prevalence in Europe, is 21–25 fractures per 1,000 children per year. To collect data, children who present to the paediatric emergency department with a suspected fracture of the distal forearm are examined using ultrasound as part of routine treatment. The aim is to rule out or diagnose a fracture. The ultrasound method is a proven non-invasive examination procedure in which an image is produced by the transmission and subsequent reception of the returning ultrasound waves, which is then assessed by a doctor. In contrast to examination modalities such as X-rays or computed tomography (CT), the person being examined is not exposed to any harmful radiation during the ultrasound procedure. In terms of patient benefit, ultrasound-based fracture diagnostics is an examination method that has been scientifically validated in recent years, particularly in children, and is used in everyday clinical practice (Douma-den Hamer et al., 2016). In particular, it has been shown that ultrasound examination is an equivalent method to X-ray examination for certain types of fracture in terms of sensitivity and specificity (radius bulge fracture, subcapital humerus fracture and clavicle fracture; Cross et al., 2010; Ackermann et al., 2020; Snelling et al., 2023).

However, it should be noted that ultrasound imaging is currently always an examiner-dependent procedure. In most cases, the acquisition and assessment of the images is performed by one person. The described user-dependency of current ultrasound diagnostics results in a decisive advantage of SonoBox: our robotic solution will make it possible for the first time to automate a standardised imaging process and thus ultimately make it person-independent.

#### 1.3.1 Ultrasound as a new modality of cross-sectional imaging

Typically, magnetic resonance imaging (MRI) and CT are regarded as representatives of 3D cross-sectional imaging. The SonoBox approach is intended to demonstrate, using the example of the diagnosis of paediatric forearm fractures, that automated ultrasound imaging is possible for the generation of comparable large-volume three-dimensional data in paediatric traumatology.

#### 1.3.2 Logistical problems of fracture diagnostics

Due to the high cost factor for purchasing and operating an appropriate X-ray system and the technical and structural safety regulations, examinations often cannot be carried out promptly and/or locally, see Schmid et al. (2017). This results in inconveniences such as waiting times or additional travelling. Furthermore, as the results of radiological examinations are only two-dimensional representations of spatial structures, ideally two images are usually taken from an angle of 90°. Occasionally, this process has to be repeated several times if the image quality is insufficient (Katzer et al., 2016).

### 1.4 Scope of this work

Development of SonoBox is still in a very early stage. As will be described below, the initial idea dates back about 10 years, when the relevant technology was not readily available. To date, a first technical prototype for phantom experiments has been constructed and is being evaluated for accuracy, reliability, and feasibility. Given that the target group for this technology, children, is considered especially vulnerable, IRB approval for studies with actual patients is under consideration but will only be given after further phantom studies will have been concluded. Consequently, this paper covers the entire process required to move from a technical idea to a first prototype, also describing steps often overlooked when medical robots are developed. These include hygiene considerations, patient and—in this case—parent experience, as well as catering to the special requirements present in an emergency room (crowded space, lack of financial means, stressed personnel).

Consequently, this work may seem to lack technical details and results, when, in fact, it strives to cover a wider range of topics instead of solely focusing on laboratory experiments without taking clinical relevance, applicability, and patient acceptance into account.

### 1.5 Current solutions for avoiding radiation exposure

In addition to the known measures to reduce radiation exposure (shielding, filters, radiation-saving device settings), the examination of bone fractures using ultrasound imaging (sonography) is currently a completely risk-free and promising alternative to the use of X-rays. In point of care ultrasound (PoCUS) examinations, the target region is imaged by trained medical staff from various standardised positions, see Ackermann et al. (2020). The transition from bone to soft tissue is shown in ultrasound images or volumes with a high contrast, so that the diagnosis of a fracture is comparable to the diagnosis using X-ray images for trained personnel. Various studies comparing radiological and sonographic examinations of bone fractures have shown that the accuracy of both methods is comparable, see Schmid et al. (2017) and Snelling et al. (2023).

However, a major advantage here is the real-time capability of the ultrasound examination, so that in case of doubt, additional data can be recorded from other angles with minimal effort.

The use of fracture sonography in adults is currently very limited, which can also be explained, for example, by more difficult examination conditions (such as the more pronounced soft tissue on the forearm). Meaning that the procedure is primarily used and recommended for children up to the age of sixteen.

Nevertheless, further studies and experiments are also proposed for the examination of adults and are already being used in remote parts of Canada and Australia, for example (Qadi et al., 2020). The great potential of the methodology to establish such procedures as a future standard is also pointed out in Champagne et al. (2019), for example. The challenges of PoCUS examinations essentially relate to the positioning of the ultrasound probe and the interpretation of the images or volumes generated. Even if the ultrasound data can be analysed with a similar degree of certainty, the forms of visualization and presentation differ massively from ultrasound or X-ray procedures. As direct contact between the probe and the patient must be ensured during these examinations, additional pressure is exerted on the affected region, which can lead to pain. This in turn can lead to the examination being made more difficult or being interrupted by movements or retraction of the arm, particularly in the context of possibly anxious and pain-ridden young patients. On the other hand, the forearm of the child patient must be held strictly in 2 planes during the X-ray examination (if necessary also by a second person), which may also mean a painful and stressful procedure.

The SonoBox project will simplify the aforementioned difficulties of positioning and interpretation in particular through the use of specialised hardware and software. As the first step of the project is to visualise children’s forearm fractures, the increased bone density in adults as well as their typically thicker layer of skin, fat, and muscle tissue is initially not critical.

### 1.6 Related works

Karlas et al. (2019) published an excellent presentation of their LEGO® robot for the automated acquisition of ultrasound images and the composition of individual images using electromagnetic tracking. The results presented show promising resolutions of the hand or even individual fingers in which the bones can be easily recognised. Furthermore, a system was developed in Ranger et al. (2019) in which a residual limb is scanned by a robotic system in a water bath with a US probe. A 3D volume was then reconstructed, which can then be used to design prosthetic sockets, for example. These publications can be seen as proof of feasibility, even if there is still a need for extensive research and development in various aspects. In particular, the setting of differentiated viewing angles and the generation of a volume of all-around images for a forearm and its corresponding positioning, the comparatively complicated tracking and considerations regarding hygiene requirements and patient safety remain unresolved. The ROPCA project at Syddansk University (DK) takes a different approach: Here, the patient’s finger joints are scanned by a robot-guided ultrasound head when arthritis is suspected or for follow-up purposes, see Frederiksen et al. (2022). This classic robotic setup requires direct contact between the probe and the patient’s body, which may not be irrelevant in terms of pain and discomfort of the patient and also in terms of general patient safety. Furthermore, the application was developed for the examination of body parts that do not need to be moved during the examination process.

Further, the development of medical devices for children is currently a neglected field of research (Tüshaus et al., 2018). Due to the complex regulations of the approval authorities in the USA and Europe, together with comparatively low case numbers in the paediatric field and the unclear market perspective, most manufacturers of medical devices are reluctant to develop variants or even new devices specially adapted to children. We have been working together for some time in the “European Paediatric Translational Research Initiative,” EPTRI for short, to overcome this dilemma (Dimitri et al., 2021).

## 2 Materials and methods

The aim of the “SonoBox” project is ultrasound-based, contact-free and automated cross-sectional imaging for the diagnosis of paediatric forearm fractures using intelligent, robotic image and data acquisition. As described above, forearm fractures and contusions are very common causes of medical consultations in children and adolescents.

As an example, the so-called Wrist SAFE (Sonographic Algorithm for Fracture Evaluation) algorithm was developed for the child’s forearm, which specifies six standard levels and a standardised procedure for risk stratification, cf. Ackermann et al. (2019). Acceptance in the medical profession for the use of ultrasound for fracture diagnostics is still underdeveloped, as finding the optimal cross-sectional images in ultrasound is particularly challenging for first-time users, requires training in the best case scenario and the documentation of the images is more complex without intelligent support. Particularly in the case of forearm fractures in children, use is often difficult, as the pressure required for imaging causes pain and in some cases the diagnosis cannot be finalised on the basis of the ultrasound image and requires a subsequent X-ray. SonoBox prototypes the development of a technology concept for the automation of data acquisition and contact-less execution of the Wrist SAFE algorithm. In the medium term, this should help to increase the efficiency of clinical processes and improve patient care. Specifically, SonoBox pursues the following goals:

This innovation is anticipated to boost the efficiency of clinical workflows and enhance patient care over the medium term. SonoBox is focused on achieving several key objectives, including reducing radiation exposure to patients and enabling point-of-care (PoC) fracture diagnostics. The technology also aims to shorten the duration of medical examinations and automate imaging processes. Future initiatives may extend to automating diagnostics. We believe that SonoBox will contribute to reduced pain for patients, which in turn can lead to higher patient compliance and comfort. Further, it could also be utilised in telemedicine.

The potentially fractured forearm is placed in a water tank in which the ultrasound probe is moved automatically and without contact with the body part to be examined in order to produce an ultrasound tomogram of the arm. SonoBox is being developed in such a way that it is able to take over the data collection from the user and provide intelligent assistance for the diagnostic evaluation. We will construct a technical platform in which a commercially available ultrasound probe is robotically guided in a water-filled tank around the extremity to be examined. With the help of fast image processing methods and intelligent robot control, we will use it to produce an ultrasound tomographic image that enables fast, painless and accurate visualisation of the possible fracture.

To perform imaging, the child’s forearm will be submerged in the water basin to allow automated scanning from all sides. Depending on the patient’s age, their injury, and level of cooperation, different methods of fixation will be required:

• No fixation: there will be a handle the child can grab and hold on to
• Slight fixation: an elastic loop is wrapped around the centre of the child’s hand (above the thumb) and attached to a hook present at the bottom of the tank
• External fixation: a second person (nurse, parent, etc.) holds the child’s arm in two places (at the hand and at the elbow)

Even though these methods need to be analysed in practise, it is expected that by not having to apply pressure to the potential fracture, the method will result in much less pain or even no pain at all.

To realise this goal, several technical challenges must be addressed. First, there is a need to enable stable, non-contact imaging to ensure that ultrasound data can be captured without direct interaction. Additionally, determining the optimal trajectory for creating tomograms—ensuring they are both fast and complete—is critical. The process must also be capable of generating tomograms with adequate spatial resolution and accuracy to meet clinical standards. Furthermore, the hygienic requirements for this technology must be clearly defined and adhered to, ensuring that the system is safe and effective for use in clinical environments.

SonoBox creates a combination of hardware and software, perspectively, can be used in perspective with any ultrasound station to visualise regions to be examined as a hazard-free volume that serves as a basis for simple diagnosis. Figure 1 shows a concept drawing.

### 2.1 Technical prototype

Based on the concept drawing shown in Figure 1, a first prototype was developed. It consists of two motorised axis to 1) laterally move the probe along the patient’s arm and 2) rotate the probe around the arm. The components of the prototype were printed in-house from polylactic acid (PLA) and, using belt drives, were connected to two brushless motors (ODrive Robotics D5065) and capacitative encoders (CUI AMT102-V). Both the motors and the encoders are controlled by the ODrive v3.6 Motor Control Board. Further components include an emergency stop switch and a USB connection to a controlling PC.

**FIGURE 2:**
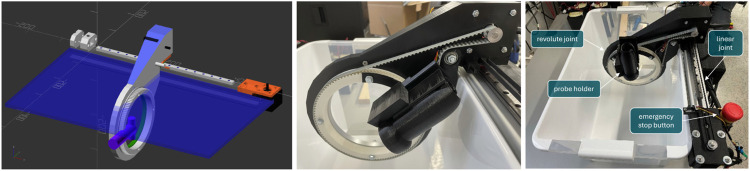
Construction of the prototype. Left: CAD model of the robotic parts, centre: details showing both belt drives and the ultrasound probe holder, right: complete prototype.

Figure 2 shows the CAD drawing used to construct the individual 3D-printed parts (left), a close-up of the belt drives as well as the probe holder (centre), and the finished prototype (right).

The robot is controlled through Python code which can directly communicate with the ODrive control board using a vendor-provided library. Consequently, it is now possible to move the robot to a specified position $p={\left(x,\theta \right)}^{\text{T}}$ , where x is the position along the linear axis ranging from 0 mm to xxx mm and θ is the angle around the rotary axis, ranging from 0° to 360°. Further, pre-programmed trajectories like helical scanning, repeated circular scanning, etc. are also possible. To make controlling the system more flexible and to integrate it with existing robotic systems, the Python code also provides an interface for integration with ROS2.

### 2.2 Safety considerations

The identification of safety and hygiene-relevant aspects for the development of SonoBox is conducted in two phases. Initially, during the requirements analysis, safety-related aspects are determined, evaluated, and incorporated into the planning process. Subsequently, during the development phase, risk management (ISO 14971) is applied, which includes an initial purpose definition and risk class determination. Repeated user tests involving typical work processes are conducted to identify safety-critical incidents and stress situations.

Developing a medical device that operates in close proximity to patients necessitates special consideration of hygiene factors. It is important to determine which materials are suitable for disinfection and what type of filling fluid (possibly with additives) can be used. Key questions include the selection of surface structures or materials that ensure ease of cleaning and patient safety, the potential use of disposable products, the assessment of infection risks associated with reuse—particularly concerning open wounds or infectious patients—and the steps necessary for product reprocessing.

Throughout the examination process, the safety of both user and patient is paramount. Potential risks are systematically analysed and evaluated using techniques like Failure Mode and Effects Analysis (FMEA). From this analysis, appropriate safety measures are derived and implemented. These measures may involve mechanical strategies, such as introducing predetermined breaking points, electronic methods, such as limiting motor currents, and software solutions like contact detection.

### 2.3 Relevance of gender and/or diversity

Boys suffer 1.2 to 1.6 times more long bone fractures than girls, especially from the age of 8. This is due to the higher risk propensity of boys during puberty and prepuberty (Mellstrand-Navarro et al., 2014).

The peak with the highest incidence of fractures occurs in girls at the age of 11 and in boys at the age of 14. On average, girls reach the end of bone growth at the age of 14 and boys at the age of 16. Based on the above information, the specific diagnosis and the resulting treatment recommendation are gender-dependent, so that gender-specific aspects are taken into account in the development work. Findings from the research field of gender HCI indicate that gender-specific results are generally possible with regard to the acceptance and usability of computer-based solutions, e.g., in usage and learning behaviour (Beckwith and Burnett, 2004). It is therefore important to evaluate the target variables “acceptance” and “usability” on a gender-specific basis to recognise any different patterns of behaviour and to be able to take these into account in the further design of the SonoBox human-machine interface.

## 3 Results

**FIGURE 3:**
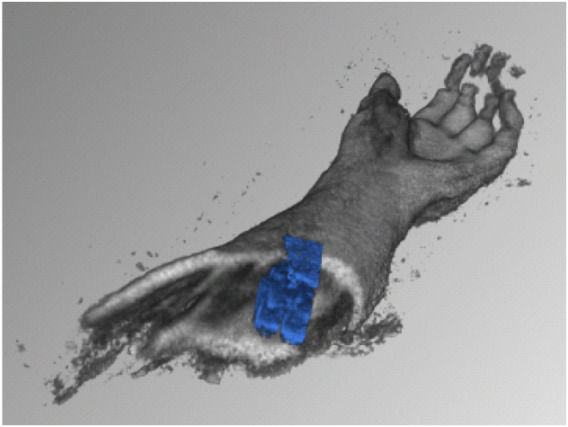
Early results of scanning a human forearm in a water tank, with fast automatic compounding of 3D ultrasound volumes [taken from Jauer et al. (2015)].

We carried out the first experiments on the rapid generation of ultrasound tomographies in a water bath in Jauer et al. (2015), see Figure 3. This combination of several ultrasound images from different positions, orientations and points in time to form an entire volume is one of our research foci. In addition to the development of fast 3D image fusion and intelligent visualisation (von Haxthausen et al., 2022), motion detection and tracking using 4D ultrasound in the context of radiotherapy is the focus of much current work: (Bruder et al., 2011; Ipsen et al., 2021; von Haxthausen et al., 2021). Depending on the speed of the analysis, motion compensation may be required in order to visualise the target volume without distortion (Ranger et al., 2019).

### 3.1 Effects of water perturbance on submerged ultrasound scanning

**FIGURE 4:**
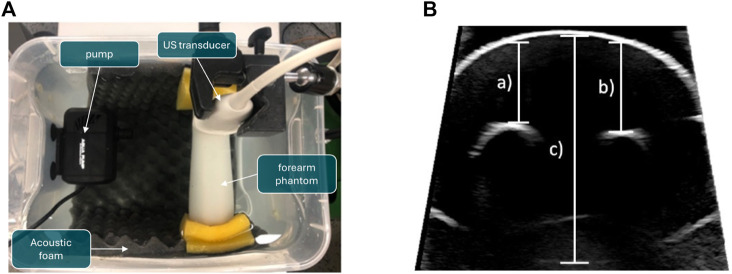
Experiment to determine the influence of turbulence in the water basin on distance measurements for compounding ultrasound data. A pump is used to swirl the water while US images of a phantom, which represents a child’s forearm, are taken. **(A)** Experimental setup. **(B)** Measured distances for validation. The resulting measurements are shown in Table 1. **(A)** Experimental setup of the water perturbation experiment. **(B)** Measured distances of the speed-of-sound experiment.

In the project, the ultrasound probe will be moved through the water while the bone is being imaged, causing water turbulence. As part of further preliminary work, it was shown that this water turbulence does not influence the quality of the ultrasound images. For this purpose, ultrasound images of a paediatric arm phantom lying in a water basin were recorded (Figure 4).

**FIGURE 5:**
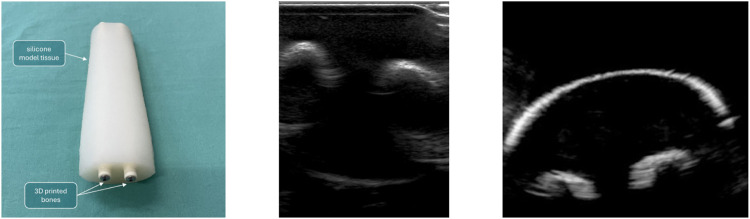
Left: Silicone-based phantom, with two embedded 3D-printed bone models. Built to mimic a child’s forearm. Centre: phantom scanned “conventionally.” Right: Phantom scanned in the water tank.

The phantom consisted of a cast silicone-based phantom with two embedded 3D-printed bone models, developed in El-Shaffey et al. (2017), and is shown in Figure 5. Further, to display quality of ultrasound images achievable under water, the phantom is imaged twice: once in a “regular” fashion, i.e., with ultrasound gel and direct contact (shown in Figure 5, centre) an once submerged in the tank and the probe inserted into the water basin (shown in Figure 5, right).

In order to simulate water turbulence, a 15 W aquarium water pump with 13 and 16 mm nozzles was used. The pump and the forearm phantom were placed at the same height under water. The water had room temperature. The phantom was clamped with sponges between the ends of a bucket. The distance between the nozzle and the forearm phantom was approximately 12 cm. The ultrasound transducer was fixed with a stand just beneath the water surface and above the forearm phantom. Ultrasound imaging was performed using a Philips EPIQ 7 ultrasound station with an L12-3 broadband linear array ultrasound transducer. In order to acquire Doppler images, a different probe (X6-1) was used, featuring a larger field of view. The bucket was further lined with acoustic foam to prevent the ultrasound from being reflected at the inner walls of the bucket.

In total, three experiments were conducted with different velocities of flow of the water pump. The following specifications were set for the three experiments:

• no water turbulence: An ultrasound image of the forearm phantom was acquired while the water wasn’t in motion.
• water turbulence: An ultrasound image of the forearm phantom was acquired with a velocity of flow of 163.74 cm/s set by the pump.
• stronger water turbulence: An ultrasound image of the forearm phantom was acquired with a velocity of flow of 313.92 cm/s set by the pump

For each setup, ultrasound images of the forearm phantom were acquired. The ultrasound station was set to the following settings: intensity gain 52%, angle 30°, depth 16 cm and frequency (data acquisition) 30 Hz.

A pump was used to generate water turbulence of varying intensity, which did not markedly affect the measurements (Table 1). This would also enable the robot to scan quickly, allowing the corresponding images to be taken in a short time.

**TABLE 1:** Resulting data of the speed-of-sound experiment. The table shows the distances measured under varying levels of disturbance in the water basin.

Speed (cm/s)	Distance a) (mm)	Distance b) (mm)	Distance c) (mm)
0	17.3	18.3	48.7
163.7	16.9	18.2	48.2
313.9	17.1	18.6	48.4

### 3.2 Reconstruction of 3D volumes

The initial prototype shown in Figure 2 was also used to reconstruct tomographic images from multiple 2D slices scanned with the aforementioned ultrasound system. Real-time access to the ultrasound data was achieved using a proprietary software library provided by Philips under NDA, coupled with the open-source PLUS toolkit (Lasso et al., 2014). For clinical purposes, we will be using the commercially available PIUR tUS Infinity system to reconstruct 3D ultrasound volumes from individual scans.

**FIGURE 6:**
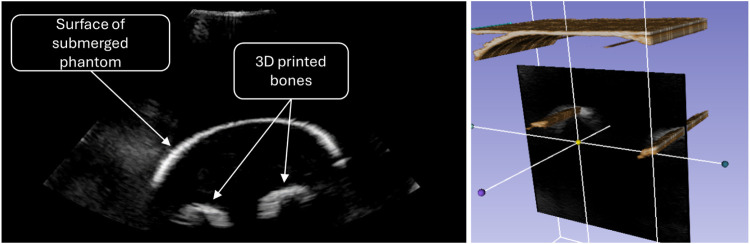
Ultrasound image of the forearm phantom (left) and volumetric reconstruction as scanned with the early prototype (right).

To collect 2D data, the robot moved the probe along the length of the phantom arm and rotated around it at the end. The data was then processed in Slicer3D (Fedorov et al., 2012), to create the reconstruction shown in Figure 6.

### 3.3 Goals of the project

The primary goal of the SonoBox project is the development and functional validation of a methodology for autonomous imaging for the diagnosis of wrist and forearm fractures in children. SonoBox is based on 3D ultrasound for a fast, cost-effective, painless and hazard-free diagnosis. The secondary objective is to extend the methodology used to other applications.

To address the technical challenges of the project, several sub-goals have been formulated. These include the construction, programming, and calibration of various prototypes, which build on preliminary work and previous efforts. The goal is to achieve fully automated imaging of the bones of the forearm and wrist by merging individual scans into a large tomographic volume, and to analyse the technical capabilities of the prototypes. Another critical sub-goal is the validation of function through a study focused on the diagnosis of distal forearm fractures in children as part of a patient study.

Additionally, the development of a hygiene concept for clinical use is essential. This involves addressing questions related to disinfection, service life, materials, water residues, and temperature. Research into possible alternatives or additives to the water and technical considerations for their implementation will also be necessary. Lastly, the project aims to develop concepts for alternative applications, leveraging the results and data from the validation study to explore various approaches for diagnosis-assisting software.

As described previously, sonography is the method of choice for examining bone fractures in children. Current research is focusing on PoCUS examinations, in which ultrasound probes are manually guided over the affected region by trained personnel. The main disadvantage of this method is the difficulty of diagnosis, which must be carried out by extensively trained personnel and—especially in children—the pressure pain caused by the examination and the resulting non-compliance of patients. Here, automated approaches can considerably simplify positioning, while 3D reconstructions of the recorded measurement data can be used to interpret an overall volume. Fast and safe movement of the probe also reduces distortions in the measurement results that can be caused by patient movement. By decoupling the ultrasound probe from the patient through the water bath, no pain is caused by pressing on the probe in the affected region. This also ensures the necessary distance from the mechanics. The hardware required for this generates additional costs, which must be set in relation to the costs of the radiological measuring equipment after considering the feasibility. However, as imaging and diagnosis are independent of each other with such an approach, the presence of a trained doctor is not required for data acquisition. The diagnosis itself can therefore be carried out independently of location and potentially be software-assisted.

## 4 Conclusion

The SonoBox initiative represents a major step forward in paediatric fracture diagnosis, offering a safer and more comfortable option compared to conventional X-ray procedures. With its automated, non-contact imaging technology, SonoBox will improve clinical workflow, reduce patient discomfort, and expand the use of ultrasound. The next phases of research and development will focus on confirming its effectiveness in clinical settings and investigating its applicability in other contexts.

Beyond fracture detection, the SonoBox’s automated analysis capabilities hold great promise for a wider range of ultrasound evaluations. These include the identification of tissue damage, nerve pathways and the analysis of vascular disease using Doppler transducers. There’s even potential for the technology to be applied to completely different fields, such as ultrasound examinations of fish in veterinary medicine. As well as setting a new standard in bone fracture diagnosis, the SonoBox project is paving the way to explore a wider range of applications and tackle more complex research questions.

## Data Availability

The original contributions presented in the study are included in the article/supplementary material, further inquiries can be directed to the corresponding author.
