# Corrigendum: SonoBox: development of a robotic ultrasound tomograph for the ultrasound diagnosis of paediatric forearm fractures

**DOI:** 10.3389/frobt.2024.1505171

**Published:** 2024-10-15

**Authors:** Floris Ernst, Jonas Osburg, Ludger Tüshaus

**Affiliations:** ^1^ Institute of Robotics and Cognitive Systems, University of Lübeck, Lübeck, Germany; ^2^ Department of Paediatric Surgery, University Hospital Schleswig-Holstein, Lübeck, Germany

**Keywords:** children, forearm fracture, ultrasound robot, radiation reduction, contactless imaging

In the published article, there was an error in the legend for **Figure 1** as published. The author of the figure was not named properly, and credit was not given for her creation. The correct legend appears below.

“A concept sketch of the envisioned SonoBox device. Figure reproduced with permission of the author Annika Dell, MSc, Fraunhofer Research Institution for Individualized and Cell-Based Medical Engineering, Lübeck, Germany.”

In the published article, there was an error in the **Acknowledgements** statement. Annika Dell was not mentioned. The correct **Acknowledgements** statement appears below.

“The authors would like to thank David Sindermann, BSc, and Jonas Kremmers, BSc, whose efforts contributed substantially to the idea of the SonoBox project and building its prototypes, as well as Annika Dell, MSc, from the Fraunhofer Research Institution for Individualized and Cell-Based Medical Engineering (IMTE) in Lübeck, whose concept sketch we were allowed to use in this publication.”

The authors apologize for these errors and state that these do not change the scientific conclusions of the article in any way. The original article has been updated.

